# Transient ALT activation protects human primary cells from chromosome instability induced by low chronic oxidative stress

**DOI:** 10.1038/srep43309

**Published:** 2017-02-27

**Authors:** Elisa Coluzzi, Rossella Buonsante, Stefano Leone, Anthony J. Asmar, Kelley L. Miller, Daniela Cimini, Antonella Sgura

**Affiliations:** 1 Department of Science, University Roma Tre, V. le G. Marconi, 446, 00146, Rome, Italy; 2 Department of Biological Sciences, Virginia Tech, Blacksburg, VA, 24061, USA; 3 Biocomplexity Institute, Virginia Tech, 1015 Life Science Circle, Blacksburg, VA, 24061, USA

## Abstract

Cells are often subjected to the effect of reactive oxygen species (ROS) as a result of both intracellular metabolism and exposure to exogenous factors. ROS-dependent oxidative stress can induce 8-oxodG within the GGG triplet found in the G-rich human telomeric sequence (TTAGGG), making telomeres highly susceptible to ROS-induced oxidative damage. Telomeres are nucleoprotein complexes that protect the ends of linear chromosomes and their dysfunction is believed to affect a wide range of cellular and/or organismal processes. Acute oxidative stress was shown to affect telomere integrity, but how prolonged low level oxidative stress, which may be more physiologically relevant, affects telomeres is still poorly investigated. Here, we explored this issue by chronically exposing human primary fibroblasts to a low dose of hydrogen peroxide. We observed fluctuating changes in telomere length and fluctuations in the rates of chromosome instability phenotypes, such that when telomeres shortened, chromosome instability increased and when telomeres lengthened, chromosome instability decreased. We found that telomere length fluctuation is associated with transient activation of an alternative lengthening of telomere (ALT) pathway, but found no evidence of cell death, impaired proliferation, or cell cycle arrest, suggesting that ALT activation may prevent oxidative damage from reaching levels that threaten cell survival.

Eukaryotic cells are constantly subjected to the effect of reactive oxygen species (ROS) as a result of both internal metabolism and external exposure (reviewed in ref. 1). The intracellular homeostasis of ROS in the body is achieved largely through anti-oxidation via an intricate antioxidant system, including both enzymatic and non-enzymatic antioxidant defenses, such as superoxide dismutase (SOD), glutathione peroxidase (GPX), catalase (CAT), glutathione (GSH), beta-carotene, vitamin A, ascorbic acid (vitamin C), and alpha-tocopherol (vitamin E)^2^.

An imbalance in redox (reduction/oxidation) regulation has been linked to uncontrolled production of ROS that results in oxidative stress^3^ and is widely recognized to damage biological molecules, thus inducing cellular toxicity^4^. Oxidative damage can act on different cellular components, such as lipids, proteins, and DNA and is implicated in aging, tumorigenesis, chronic inflammation^5^, neurodegeneration, and chemical toxicity (reviewed in refs 6 and 7). The main type of DNA damage induced by oxidative stress is the modification of DNA bases to species such as 8-oxo-guanine (8-oxoGua), thymine glycol, and 5-hydroxy-methyluracil. Furthermore, because of their ability to induce both single and double strand DNA breaks^8^,^9^, high levels of ROS may explain some aspects of the genomic instability^10^,^11^ associated with tumorigenesis^12^,^13^.

Previous studies have shown that telomeres are highly susceptible to oxidative damage^14^,^15^. Telomeres are nucleoprotein complexes that protect the ends of linear chromosomes and their dysfunction has been linked to a wide range of cellular and/or organismal processes, including apoptosis, aging, chromosomal instability, and cancer^16^,^17^,^18^,^19^,^20^. The ability of ROS to induce 8-oxodG within the GGG triplet found in the G-rich human telomeric sequence (TTAGGG)^14^,^21^ can explain why telomeres are particularly susceptible to oxidative stress-induced damage. This effect may be further enhanced by the inefficiency of DNA repair within telomeric chromosome regions compared to the rest of the genome^22^,^23^,^24^. It was previously shown that acute oxidative stress accelerates telomere shortening^24^,^25^,^26^. Moreover, many studies have investigated the effects of radiation exposure and it has been suggested that some of the observed effects are caused by ROS generated as a by-product of radiation exposure (reviewed in ref. 27). However, in such studies it is difficult to separate the direct effects of radiation from the secondary effects caused by ROS. Thus, the effects of low, chronic oxidative stress on telomere metabolism remain poorly investigated. Specifically, it is not known whether prolonged low levels of oxidative stress, which may better reflect the levels of oxidative stress cells are challenged with in their native environment, may be sufficient to produce a physiologically relevant effect on telomere stability.

In this study, we investigated this issue in human primary fibroblasts exposed to low levels of oxidative stress over prolonged periods of time. Our findings revealed a telomere-specific effect that resulted in the emergence of chromosome instability phenotypes and transient activation of an alternative lengthening of telomere (ALT) pathway.

## Results

To investigate the effects of low levels of chronic oxidative stress over prolonged periods of time, we treated human primary lung fibroblasts (MRC-5 cells) daily with 10 μM H_2_O_2_. MRC-5 cells represent a good model given that they are primary, non-transformed, non-immortalized, telomerase negative cells, with functional cell cycle checkpoints, and thus expected to display a response to oxidative damage that reflects the response of healthy human cells. For this study, H_2_O_2_-treated and untreated MRC-5 cells were analyzed periodically over a period of 20 days.

### Prolonged low-level oxidative stress induces telomere length changes

**Figure 1:**
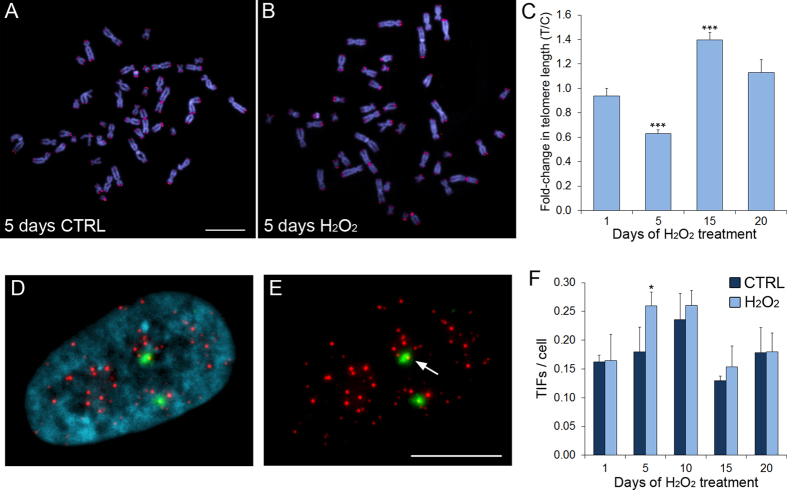
Prolonged low-level oxidative stress induces fluctuating changes in telomere length. (**A**) Telomere FISH staining of control MRC-5 cell metaphase spread. (**B**) Telomere FISH staining of metaphase spread from MRC-5 cells treated with 10 μM H_2_O_2_ for 5 days. In (**A** and **B**) DNA is shown in blue, whereas telomere and chromosome 2 centromere staining is shown in red. Scale bar, 5 μm. (**C**) Quantification of the change in T/C ratio (see Materials and Methods for details on Q-FISH analysis) in H_2_O_2_-treated cells compared to control cells. The normalized (over control) data are reported as mean ± s.e.m. Asterisks denote statistical significance (Mann Whitney U test, ***p < 0.0001). (**D**,**E**) Telomere dysfunction-induced foci (TIFs) in nucleus of MRC-5 cell. DNA is shown in blue, telomeres in red, and γ-H2AX foci in green. Arrow in (**E**) points to instance of γ-H2AX/telomere colocalization (TIF). Scale bar, 5 μm. (**F**) Quantification of TIFs. The data are reported as mean ± s.e.m. Asterisk denotes statistical significance (Fisher’s exact test, *p = 0.05).

To test whether prolonged oxidative stress specifically affected telomeres, we measured telomere length by Q-FISH (Quantitative Fluorescent *in Situ* Hybridization) on MRC-5 metaphase spreads (Fig. 1A,B). We found that daily H_2_O_2_ treatment caused telomere shortening after 5 days of treatment, which was followed by significant lengthening at 15 days (Figs 1C and S1).

To determine whether the change in telomere length was caused by H_2_O_2_-induced DNA damage specifically at telomeres, we performed immuno-FISH by combining immunofluorescence staining for phosphorylated H2AX (γ-H2AX) and telomere FISH staining (Fig. 1D,E) and quantified sites of co-localization, known as telomere dysfunction-induced foci (TIFs). We found that at the 5 day time point, the H_2_O_2_-treated cells displayed significantly higher numbers of TIFs compared to the untreated (CTRL) cells (Fig. 1F), supporting the idea that the telomere shortening observed after 5 days of H_2_O_2_ treatment (Fig. 1C) can arise from accumulation of DNA damage at telomeres.

### Prolonged low-level oxidative stress induces anaphase chromosome bridges and abnormal nuclear morphologies

**Figure 2:**
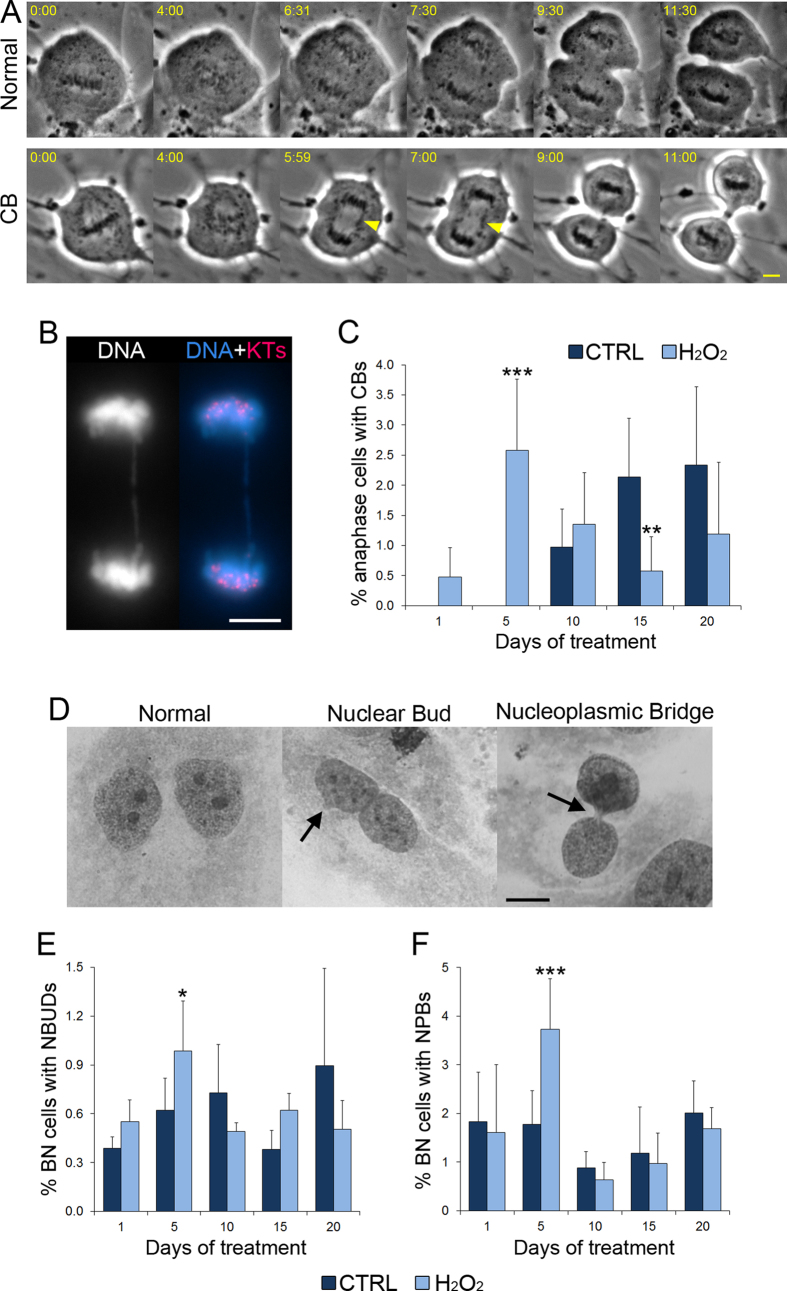
Prolonged low-level oxidative stress induces anaphase chromosome bridges and abnormal nuclear morphologies. (**A**) Still images from phase contrast microscopy time-lapse videos of MRC-5 cells undergoing mitosis and displaying normal chromosome segregation (top) or an anaphase chromosome bridge (CB, bottom). The second frame in each series represents the onset of chromosome segregation. The elapsed time is indicated in min:sec. Arrowheads point at the CB. Scale bar, 5 μm. (**B**) Fixed and fluorescently stained anaphase MRC-5 cell displaying a CB. DNA alone is shown in the left panel, whereas the right panel shows an overlay of DNA (blue) imaged at a single focal plane with a maximum fluorescence intensity projection of kinetochore (KT) staining (red). Scale bar, 5 μm. (**C**) Cumulative data from live- (**A**) and fixed-cell (**B**) experiments reporting the frequencies of untreated (CTRL) and 10 μM H_2_O_2_-treated anaphase cells with chromosome bridges (CBs). The data are reported as mean ± s.e.m. Asterisks denote significance (Fisher’s exact test, **p < 0.01; ***p < 0.001) for comparison of data from H_2_O_2_-treated cells with corresponding controls (CTRL). (**D**) Giemsa-stained binucleate (BN) cells from cytokinesis-block assay displaying normal nuclear morphology (left panel), a nuclear bud (middle panel, arrrow), and a nucleoplasmic bridge (right panel, arrow). Scale bar, 5 μm. (**E**,**F)** Frequencies of BN cells displaying nuclear buds (NBUDs, **E**) or nucleoplasmic bridges (NPBs, **F**). The data are reported as mean ± s.e.m. Asterisks denote statistical significance (χ^2^, *p < 0.05; ***p < 0.001) for comparison of data from H_2_O_2_-treated cells with corresponding controls (CTRL).

One acknowledged consequence of telomere shortening is the end-to-end fusion of chromosomes with eroded telomeres, which leads to the formation of chromosome bridges (CBs) during anaphase^28^. To test whether the H_2_O_2_-induced changes in telomere length were accompanied by changes in the rates of CBs, we analyzed both live cells undergoing mitosis to identify chromosome bridges as cells underwent anaphase (Fig. 2A) and fixed anaphase cells immunostained for kinetochore proteins (Fig. 2B). We found that the frequencies of CBs in H_2_O_2_-treated cells increased at the 5 day time point, but then dropped by 15 days (Fig. 2C).

We next evaluated the presence of abnormal nuclear structures in a cytokinesis-block assay (Fig. 2D), which allows the products of a single mitosis to be retained in the same cytoplasm^29^. We determined the frequencies of nuclear buds (NBUDs, protrusions of the nucleus; Fig. 2D, middle) and nucleoplasmic bridges (NPBs, chromatin bridges between two fully re-formed nuclei; Fig. 2D, right). NBUDs can represent the remnants of a broken CB, whereas NPBs represent unbroken CBs that persist beyond the completion of mitosis^30^. Consistent with this, we found that there was an increase in the frequencies of both NBUDs (Fig. 2E) and NPBs (Fig. 2F) in H_2_O_2_-treated cells at the 5 day time point, followed by a decrease at later time points.

Interestingly, the fluctuations in telomere length (Fig. 1C) displayed an inverse pattern compared to the fluctuations observed for the frequencies of CBs (Fig. 2C), NBUDs (Fig. 2E), and NPBs (Fig. 2F) in H_2_O_2_-treated cells. Indeed, the initial increase in CBs, NBUDs, and NPBs correlated with telomere shortening (see Figs 1C and 2C,E,F, 5 days), whereas the subsequent decrease in CBs, NBUDs, and NPBs correlated with telomere lengthening (see Figs 1C and 2C,E,F, 15 days), suggesting that the changes in telomere length are responsible for the observed CBs, NBUDs, and NPBs.

### Prolonged exposure to hydrogen peroxide does not affect cell proliferation or cell cycle progression

**Figure 3:**
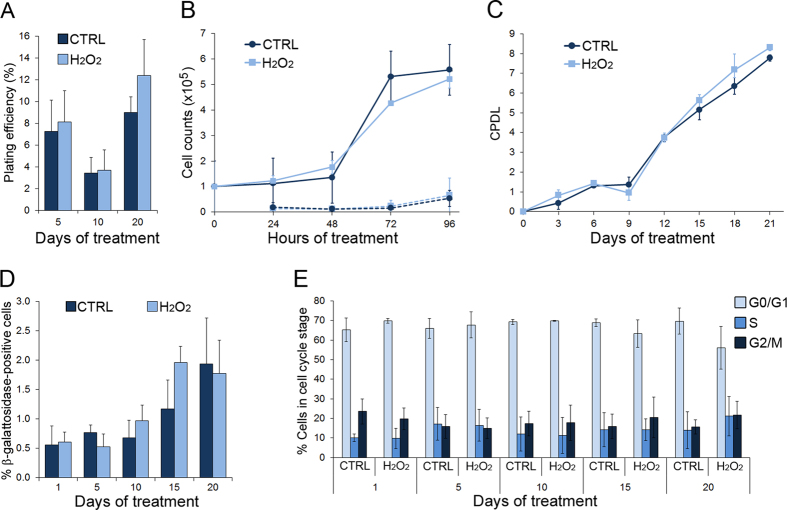
Prolonged exposure to hydrogen peroxide does not affect cell proliferation or cell cycle progression. (**A**) Plating efficiency from colony formation assays in untreated (CTRL) and H_2_O_2_-treated cells. The data are reported as mean ± s.e.m. (**B**) Quantification of live (solid lines) and dead (dashed lines) cells from flow cytometric analysis (volumetric absolute counting) performed over a four-day period (96 hours). Data are reported as mean ± s.d. (**C**) Cumulative population doubling level (CPDL) in untreated (CTRL) and H_2_O_2_-treated cells over a 21-day period. Data are reported as mean ± s.d. (**D**) Frequencies of β-galactosidase (senescence marker)-positive cells in untreated (CTRL) and H_2_O_2_-treated cells. Data are reported as mean ± s.e.m. (**E**) Data from FACS analysis showing proportion of cells in different phases of the cell cycle in untreated (CTRL) and H_2_O_2_-treated samples. Data are reported as mean ± s.d.

We next performed a number of assays to assess whether the fluctuations in telomere length and frequencies of CBs, NBUDs, and NPBs were caused by cell death, cell cycle arrest, or cell cycle delay of a sub-population of cells with short telomeres, which would lead to depletion of cells with shorter telomeres (and hence cells with CBs, NBUDs, and NPBs) from the dividing cell pool. First, we performed a colony formation assay to determine whether the H_2_O_2_-treated cells displayed reduced survival following treatment compared to corresponding untreated cells. We found that the colony formation efficiency of H_2_O_2_-treated cells was comparable to that of untreated cells (Fig. 3A) at all time points analyzed. We then used flow cytometric analysis of unfixed samples stained with propidium iodide (PI) to perform cell counts over a 96 hour period. Volumetric absolute counting was used for cell enumeration and PI-positive cells (dead cells) were excluded from the counts of live cells. The data showed very small numbers of dead cells in both the untreated (CTRL) and the H_2_O_2_-treated cell populations at all time points analyzed (Fig. 3B, dashed lines). Conversely, live cells represented the vast majority of both the untreated (CTRL) and the H_2_O_2_-treated cell populations (Fig. 3B, solid lines). Moreover, our data showed that cell proliferation occurred at similar rates in the untreated and the H_2_O_2_-treated cell populations over the four-day period (Fig. 3B, solid lines). To determine whether the two populations maintained similar proliferation rates over time, we measured cumulative population doublings over a three-week period (see Materials and Methods for details) and again found no difference in population doubling rates between the untreated (CTRL) and the H_2_O_2_-treated cell populations (Fig. 3C). Next, we performed a β-galactosidase assay to assess whether any significant fraction of the two cell populations was reaching senescence and therefore exiting the cell cycle. We found that although there was a slight increase in the number of senescent cells over time in both H_2_O_2_-treated and untreated (CTRL) cell populations, the number of senescent cells remained small (≤2%) throughout the experiment and there was no significant difference between H_2_O_2_-treated and untreated (CTRL) cells (Fig. 3D). Our β-galactosidase data differ from those of previous studies^31^,^32^, which found that prolonged oxidative stress induced senescence associated with changes in telomere length. The different results in one of these studies may be explained by the use of a different cell type and a different treatment regime^31^. The inconsistency with the other study^32^ is more difficult to explain. However, here we present further evidence that senescence-dependent cell cycle arrest did not occur. Indeed, we found no changes in cell proliferation (Fig. 3A–C). Moreover, we further confirmed lack of senescence-dependent G1 arrest by flow cytometric analysis, which showed only minor variations in the distribution of cells at different cell cycle stages and no obvious arrest/delay at any particular stage when comparing H_2_O_2_-treated and untreated (CTRL) samples (Fig. 3E).

Overall, our data indicate that the increase in average telomere length observed at 15 days of H_2_O_2_ treatment was not due to elimination of cells with short telomeres from the mitotic cell population due to reduced proliferation, cell death, senescence, and/or cell cycle arrest.

### Evidence of ALT-dependent telomere lengthening in cells under prolonged low-level oxidative stress

Having excluded cell cycle arrest, senescence, and/or cell death as a cause of the increase in average telomere length observed after 15 days of H_2_O_2_ treatment, we postulated that cells under oxidative stress were able to activate some mechanism of telomere elongation. Thus, we set out to identify the mechanism responsible for telomere length modulation in cells under low levels of chronic oxidative stress.

**Figure 4:**
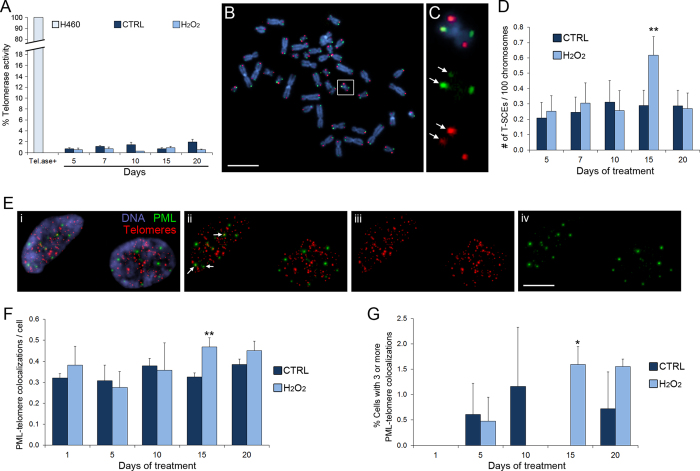
Prolonged low-level oxidative stress results in activation of ALT-dependent telomere lengthening. (**A**) Data from RTQ-TRAP assay showing lack of telomerase activity in MRC-5 cells in response to H_2_O_2_-induced low-level oxidative stress. This is evidenced by the very low levels of telomerase activity observed in both H_2_O_2_-treated and untreated (CTRL) MRC-5 cells compared to the levels of telomerase activity observed in the telomerase-positive H460 cells. (**B**) CO-FISH staining of metaphase spread from MRC-5 cell. DNA is shown in blue, whereas the TTAGGG and CCCTAA telomeric sequences are shown in red and green, respectively. Scale bar, 5 μm. (**C**) Enlarged (400%) view of the chromosome from the boxed region in (**B**), showing a telomeric sister chromatid exchange (T-SCE). Overlay of all three colors is shown in the top panel; images of staining with individual CO-FISH probes are shown in the middle and bottom panels. Arrows point at signals on both sister chromatids (evidence of T-SCE). (**D**) Data from CO-FISH analysis reporting the number of T-SCEs over 100 chromosomes. The data are reported as mean ± s.e.m. Asterisks denote statistical significance (binomial probability test, **p < 0.05) when data from H_2_O_2_-treated cells were compared to corresponding control (CTRL) data. (**E**) Nuclei (blue) of MRC-5 cells displaying combined PML immunostaining (green) and telomere FISH (red). The four panels, from left to right display, respectively, overlay of all three colors (i), overlay of PML and telomere staining (ii), telomere staining alone (iii), and PML staining alone (iv). Arrows in (ii) point at colocalization of PML and telomere signals. Scale bar, 5 μm. (**F**) Data from analysis of PML-telomere colocalization, in which the absolute number of colocalizations/nucleus is reported. The data are reported as mean ± s.e.m. Asterisks denote statistical significance (Fisher’s exact test, **p < 0.05) when data from H_2_O_2_-treated cells were compared to data from corresponding controls (CTRL). (**G**) Frequencies of colocalization-positive cells (3 or more PML-telomere colocalizations). The data are reported as mean ± s.e.m. Asterisk refers to statistical test result (Fisher’s exact test, *p < 0.1) when data from H_2_O_2_-treated cells were compared to corresponding control (CTRL) data.

It is widely acknowledged that cells can elongate telomeres by either telomerase-dependent or telomerase-independent mechanisms^33^,^34^. Thus, we first performed a RTQ-TRAP (Real-Time Quantitative Telomeric Repeat Amplification Protocol) assay to measure telomerase activity over the 20 days of H_2_O_2_ treatment. We did not find any evidence of telomerase activity at any of the time points analyzed for either the untreated (CTRL) or the H_2_O_2_-treated samples (Fig. 4A), suggesting that the observed telomere elongation (Fig. 1C) may occur via a telomerase-independent mechanism.

Telomerase-negative cancer cells were shown to activate a recombination-based mechanism of telomere maintenance known as Alternative Lengthening of Telomeres (ALT)^35^. To test whether the telomere elongation we observed at 15 days of H_2_O_2_ treatment was the result of ALT pathway activation, we (i) employed CO-FISH (Chromosome Orientation FISH), which can detect recombination events between sister telomeres (or Telomere Sister Chromatid Exchanges, T-SCEs; Fig. 4B,C) and (ii) assessed the co-localization of telomeres with the promyelocitic leukemia (PML) protein (Fig. 4E), which was previously shown to serve as a marker of telomerase-independent telomere lengthening^36^. We found that at the 15-day time point, H_2_O_2_-treated cells displayed sister telomere exchanges at frequencies that were significantly higher than those observed in untreated cells (Fig. 4D), but this difference disappeared at the subsequent time point (Fig. 4D). Consistently, telomere-PML colocalization occurred at significantly higher rates in H_2_O_2_-treated cells compared to untreated (CTRL) cells at the 15 day time point (Fig. 4F,G). These observations are also consistent with the increase in telomere length heterogeneity (another hallmark of ALT) observed in H_2_O_2_-treated cells at the 15-day time point, as indicated by the marked increase in telomere length variance (Figure S1). Taken together, these data indicate that human primary fibroblasts can transiently activate an ALT mechanism for telomere maintenance in response to low chronic oxidative stress.

## Discussion

In this study, we investigated the effects of chronic, low levels of oxidative stress on telomeres of human primary fibroblasts. We found that initial telomere shortening, was followed by telomere elongation (Fig. 1A–C), indicating that the cell can activate mechanisms to cope with this exogenous challenge. Our data also showed that the telomere elongation depended on ALT rather than telomerase re-activation (Fig. 4), indicating that activation of the ALT mechanism is not exclusive to cancer cells.

### Prolonged oxidative stress results in fluctuating changes in telomere length and chromosome instability phenotypes

**Figure 5:**
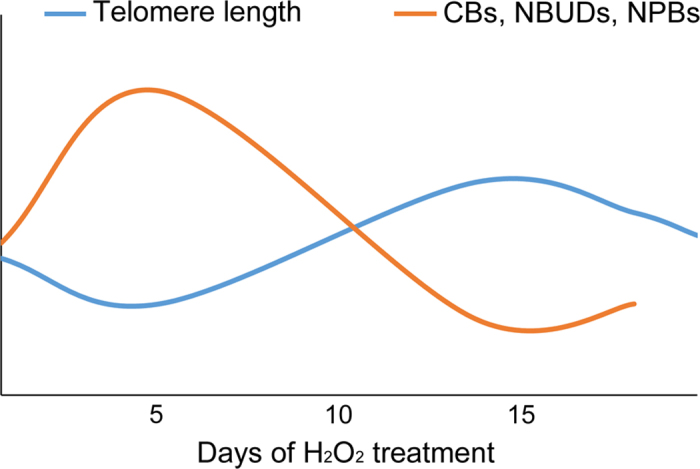
Diagrammatic summary of the results from the present study. The data presented in this study show that prolonged low levels of oxidative stress induce fluctuating changes in telomere length in human primary fibroblasts. The frequencies of chromosome instability markers (CBs, NBUDs, NPBs) that typically arise as a result of telomere fusions also display fluctuating changes. However, these changes exhibit an inverse correlation with the changes in telomere length, suggesting that as telomeres shorten, they fuse and cause the formation of chromosome bridges (CBs), which in turn can lead to the formation of nuclear buds (NBUDs) and nucleoplasmic bridges (NPBs). Indeed, our data show that as telomeres length decreases, CBs, NBUDs, and NPBs increase, whereas when telomeres length increases, CBs, NBUDs, and NPBs decrease. Interestingly, activation of the ALT pathway was found to coincide with telomere lengthening at the 15 day time point (see Fig. 4D).

Previous studies had shown a link between oxidative stress and telomere shortening^24^,^26^. However, such studies used acute treatment regimens (e.g., high doses of H_2_O_2_ for short times). Here, we find that prolonged, low levels of oxidative stress can also result in telomere shortening. However, we found that telomere length underwent fluctuating changes, by which telomere shortening was followed by telomere elongation, and these changes inversely correlated with CBs, NBUDs, and NPBs, so that when telomeres reached their shortest length, we observed an increase in CBs, NBUDs, and NPBs (see diagram in Fig. 5). Importantly, CBs, NBUDs, and NPBs can all arise as a result of telomere fusions^30^,^37^, and are thus expected to rise as telomeres shorten and decrease as telomeres lengthen. The fluctuations in telomere length are an interesting and novel result. Increases in telomere length were previously observed at late time points after treatment of human primary fibroblasts with X-ray radiation or low-energy protons^38^,^39^. Although these previous studies did not report fluctuations in telomere length, the observation that both radiations and oxidative damage can trigger telomere lengthening suggests that radiations may promote telomere lengthening through their ability to induce oxidative stress. Thus, our data suggest that, even if induced by different agents, oxidative stress can activate common pathways that compensate telomere shortening and lead to a fluctuation in telomere length.

It should also be noted that the frequencies of CBs, NBUDs and NPBs never reached very high levels (e.g., up to 60% of cells can display CBs as a result of telomere dysfunction or DNA double strand breaks^40^, whereas in the present study these frequencies were only a few percent). Moreover, by using numerous assays, we found no evidence of cell death or cell cycle arrest. Taken together, these findings suggest that activation of telomere lengthening pathways may occur before the telomere damage-induced chromosomal instability reaches levels that could result in cell death and affect the overall health of the cell population.

### Fluctuations in telomere length arise as a consequence of transient ALT activation

Our data show that the telomere lengthening that follows the initial shortening is not due to telomerase activation but to induction of the ALT pathway. ALT-mediated elongation of telomeres is associated with recombination events between sister chromatids within the telomeric region, a process known as telomeric sister chromatid exchange (T-SCE)^41^. It has also been shown that a hallmark of ALT activation is the presence of ALT-associated promyelocytic leukemia nuclear bodies (APBs), which are promyelocytic leukemia nuclear bodies (PNBs) containing telomeric material as well as a host of DNA repair, replication, and recombination factors^36^. ALT activation has been reported to occur in several abnormal situations, including human tumors, immortalized human cell lines, and telomerase-null mouse cells^42^,^43^. Here, we present evidence of ALT activation in human primary fibroblasts. This is interesting because it indicates that ALT activation, which not only ensures telomere lengthening but can also lead to immortalization, is not exclusive to transformed cells or cells with telomere-impairing genetic mutations. Activation of the ALT pathway in human primary fibroblasts was previously observed in response to high-LET radiation^39^. The fibroblasts used in this earlier study were of different origin (foreskin) compared to the ones used here (lung), indicating that the ability to activate the ALT pathway may be general among human fibroblasts.

An interesting observation of our study is that the ALT pathway does not persist in an activated state, but is activated transiently (Fig. 4). This was also the case in human primary fibroblasts exposed to high-LET radiation^39^. Based on this observation, this earlier study proposed that ALT activation, as opposed to telomerase re-activation, may be viewed as an emergency mechanism, which gets activated only when DNA damage becomes severe, but is silenced again when the damage is rescued to levels that allow cell survival^39^. However, in the present study we found ALT activation, but no evidence of cell death or cell cycle arrest/delay, indicating that the ALT pathway can be activated even in the presence of mild, rather than severe, damage. Indeed, although ALT activation was found at a time when telomeres were shortest and the frequencies of CBs, NBUDs, and NPBs were highest (Fig. 5), as discussed in the previous section, these defects never reached very high levels. This suggests that ALT activation may occur as the damage increases, but before the damage becomes so severe to threaten cell survival and/or integrity of the genome. Although ALT mechanisms were believed for a long time to occur exclusively in cancer cells or in cells with impairing genetic mutations, it is perhaps not surprising that even non-transformed cells can exploit this pathway. In fact, being a recombination-based mechanism, cells may have many of the required enzymes (e.g., ATR, RPA) readily available, as these enzymes are typically used by the cell for homologous recombination-based DNA repair. Conversely, re-activation of telomerase activity would not only require synthesis of an enzyme that is typically not produced by normal cells, but may put the cell at risk of acquiring immortality, which is the first step towards transformation. Indeed, there is no evidence so far that telomerase may be turned off once it is re-activated. Conversely, the ability of the ALT pathway to be turned on and off as needed allows the cell to respond to external challenges quickly without acquiring an immortal phenotype. An interesting question for the future is whether fibroblasts may display a lower propensity compared to epithelial cells to activate telomerase over the ALT pathway in response to external challenges and/or DNA damage. Such a preference could, in part, explain the resistance of fibroblasts to immortality and transformation compared to epithelial cells.

## Materials and Methods

### Cell culture conditions and treatment

MRC-5 cells (fetal human lung primary fibroblasts; ECACC, UK) were grown in modified Eagle’s medium (MEM) (Euroclone) supplemented with 10% FBS, 10,000 units/ml penicillin and 10 mg/ml streptomycin (Biological Industries), 1% L-Glutamine, and 1% non-essential aminoacids, and were maintained at 37 °C in a humidified incubator with 5% CO_2_. For experiments, cells were grown either in 25 cm^2^ flasks or on sterilized coverslips inside 35 mm Petri dishes and treated daily with 10 μM H_2_O_2_ (10 vol, 3%). The cells were split and passaged when they reached confluency. Thus, treatments were performed on diving cell cultures. The cells were analyzed at regular time intervals over a period of 20 days, and cells treated with H_2_O_2_ were compared to parallel control cells grown under the same conditions, but in medium without H_2_O_2_. H_2_O_2_-containing (treated) or plain (control) medium was replaced daily. Although cells were treated for many consecutive days, all data analysis was performed on cells that had been split ~50 hours before, which corresponds to the time when the cell population appears to undergo doubling (see Fig. 3B).

### Preparation of metaphase spreads

For metaphase spreads, cells in 25 cm^2^ flasks were pre-incubated for 5 hrs with 5 × 10^−6^ M colchicine (Sigma Aldrich, St. Louis, USA). Metaphase spreads were prepared following a standard procedure consisting of treatment with hypotonic solution (75 mM KCl) for 28 min at 37 °C, followed by fixation in freshly prepared fixative (3:1 v/v methanol:acetic acid). Cells were then dropped onto microscope slides, air dried, and utilized for Q- and CO-FISH analysis.

### Quantitative-FISH Analysis (Q-FISH)

Q-FISH staining was performed as previously described^44^, with minor modifications. Briefly, 48 hrs after preparation of metaphase spreads, slides were rinsed in PBS, fixed in 4% formaldehyde for 2 min and rinsed again in PBS. The slides were then incubated in acidified pepsin solution for 10 min, rinsed in PBS and fixed again in 4% formaldehyde for 2 min. After further rinses, the slides were dehydrated through an ethanol series (70%, 80%, and 100%). Cy3-labeled telomeric (Panagene, Korea) and chromosome 2 centromeric (DAKO Cytomatation, Denmark) Peptide Nucleic Acid (PNA) probes were used for hybridization. Slides and probes were co-denatured at 80 °C for 3 min and hybridized for 2 hrs at room temperature in a humidified chamber. After hybridization, slides were washed twice for 15 min in 70% formamide, 10 mM Tris pH 7.2, and 0.1% BSA, followed by three washes in TBS/Tween 0.08% (0.1 M Tris pH 7.5, 0.15 M NaCl, 0.08% Tween 20). Slides were then dehydrated through an ethanol series and air-dried. Finally, slides were counterstained with 4,6-diamidino-2 phenylindole (DAPI; Sigma Aldrich, St. Louis, USA) in Vectashield (Vector Laboratories, CA, USA). Images were captured with an Axio Imager M1 microscope (Carl Zeiss, Germany) equipped with a 63x objective and a Cool Cube 1 CCD camera (MetaSystems, Germany). Telomere size was determined with the ISIS software (MetaSystems, Germany). The software calculates telomere lengths as the ratio (T/C) between the total telomere fluorescence (T) and the fluorescence of the centromeres of the two chromosomes 2 (C)^45^, which is used as the internal reference in each metaphase spread analyzed. The data are reported as the average of 10–15 metaphase spreads (i.e., 1,756 or more telomeres) per data point from each of 2 independent experiments.

### TIF immuno-FISH staining

Cells were seeded on glass coverslips inside 35 mm Petri dishes and kept in the incubator until fixation. Cells were fixed with 4% paraformaldehyde (Sigma Aldrich, St. Louis, USA), then permeabilized with 0.2% Triton X-100, and blocked in 1% BSA in PBS for 30 min at room temperature. Samples were incubated overnight at 4 °C with a mouse mono-clonal anti-phospho-histone H2AX antibody (γ-H2AX; Millipore, Temecula, CA, USA) diluted 1:100. Coverslips were then washed in 0.05% Triton X-100/PBS and incubated for 1 hr at 37 °C with an Alexa 488-labelled donkey anti-mouse antibody (Invitrogen, Life Technologies, Carlsbad, CA, USA) diluted 1:200. After washes in 0.05% Triton X-100/PBS, the coverslips were fixed again in 4% formaldehyde for 2 min and dehydrated through an ethanol series (70, 80, 100%). Coverslips and probe (Cy3 linked telomeric PNA probe; DAKO Cytomatation, Denmark) were co-denatured at 80 °C for 3 min and hybridized for 2 hrs at room temperature in a humidified chamber. After hybridization, the coverslips were washed twice for 15 min in 50% formamide, 10 mM Tris, pH 7.2, and 0.1% BSA, followed by three 5 min washes in 0.1 M Tris, pH 7.5, 0.15 M NaCl, and 0.08%Tween 20. The coverslips were then dehydrated by immersion through an ethanol series and air dried. Finally, the coverslips were counterstained with DAPI (Sigma Aldrich, St. Louis, USA) in Vectashield (Vector Laboratories, Burlingame, CA) and mounted on microscope slides. Images were acquired using an Axio Imager M1 microscope (Carl Zeiss, Germany) equipped with a 63x objective and a Cool Cube 1 CCD camera (MetaSystems, Germany). Images were then analyzed to identify instances of colocalization between γ-H2AX foci and telomere signals (telomere dysfunction-induced foci, TIFs). The number of TIFs per cell was determined by analyzing 100 cells from each of at least two independent experiments.

### Time-lapse microscopy and analysis of chromosome bridges in live mitotic cells

Cells were seeded on acid-washed, sterile coverslips inside 35 mm Petri dishes. Coverslips at ~70% confluence were mounted into a Rose chamber^46^ without top coverslip. The chamber was filled with L-15 medium with 4.5 g/L glucose, and mineral oil was added on top to prevent evaporation. Imaging was performed on a Nikon Eclipse TE2000-U inverted microscope (Nikon Instruments Inc., NY, USA) equipped with phase-contrast trans-illumination, transmitted light shutter, ProScan automated stage (Prior Scientific, Cambridge, UK), and HQ2 CCD camera (Photometrics, AZ, USA). Cells were maintained at ~36 °C by means of an air stream stage incubator (Nevtek, VA, USA). Image acquisition, light shutter, and stage position were controlled by NIS Elements AR software (Nikon Instruments Inc., NY, USA) on a PC computer. Images of ten or more different fields of view were acquired at 1 minute intervals over a three-hour period with a Plan Fluor ELWD 40x/, 0.6 NA Ph2 ALD objective (Nikon Instruments Inc., NY, USA). The time-lapse movies were subsequently played back to visualize cells undergoing mitosis during the period of recording and identify cells displaying chromosome bridges during anaphase. The experiment was repeated twice and a total of 48–111 mitotic cells were analyzed per experimental point.

### Kinetochore immunostaining and analysis of chromosome bridges in fixed cells

Cells on coverslips inside 35 mm Petri dishes were rapidly rinsed in PBS, fixed for 20 min in 4% formaldehyde, washed in PBS 3 times, and then permeabilized for 10 min in PHEM buffer (60 mM PIPES, 21 mM HEPES, 10 mM EGTA, 2 mM MgCl_2_, 685 mM NaCl) containing 0.5% Triton X-100. Subsequently, cells were washed in PBS 3 times, and then blocked in 10% boiled goat serum for 1 hr at room temperature. The coverslips were then incubated overnight at 4 °C in primary antibody diluted in 5% boiled goat serum. Cells were next washed in PBST (PBS with 0.05% Tween 20), incubated in secondary antibodies diluted in 5% boiled goat serum for 1 hr at room temperature, washed in PBST, stained with DAPI, washed again, and mounted in an antifade solution containing 90% glycerol and 0.5% N-propyl gallate. The primary antibody, human ACA (anti-centromere antibodies, derived from human CREST patient serum; Antibodies Inc., CA, USA), was diluted 1:100 and the secondary antibody, X-Rhodamine goat-anti-human (Jackson ImmunoResearch Laboratories, Inc., PA, USA), was diluted 1:100.

Representative examples of immunofluorescently stained cells were imaged with a Swept Field Confocal system (Prairie Technologies, WI, USA) on a Nikon Eclipse TE2000-U inverted microscope (Nikon Instruments Inc., NY, USA) equipped with a 100X/1.4 NA Plan-Apochromatic phase-contrast objective lens (Nikon Instruments Inc., NY, USA) and automated ProScan stage (Prior Scientific, Cambridge, UK). The confocal head was equipped with filters for illumination at 405, 488, 561, and 640 nm from a 4 channel high power laser combiner launch (Agilent Technologies, CA, USA). Digital images were acquired with an HQ2 CCD camera (Photometrics, AZ, USA). Image acquisition, Z-axis position, laser lines, and confocal system were all controlled by NIS Elements AR software (Nikon Instruments Inc, NY, USA) on a PC computer. Z-series optical sections through each cell analyzed were obtained at 0.6 μm steps. Frequencies of chromosome bridges were determined by viewing the samples via appropriate filter sets (Chroma Technologies, VT, USA) on the same microscope. The data represent the average of 3 independent experiments in which a total of 100–200 anaphases per data point were analyzed.

### Cytokinesis-block assay and analysis of NBUDs and NPBs

Binucleate (BN) MCR-5 cells were obtained by incubating the cells in 3 μg/ml cytocalasin B (Sigma Aldrich, St. Louis, USA) during the 24 hrs preceding fixation. Cells were fixed in freshly prepared fixative (5:1 v/v methanol:acetic acid) and subsequently stained in 5% Giemsa (BDH Laboratories Supplies, Poole, England) in water. Cells were considered BN when the distance between the two daughter nuclei was not greater than the radius of one nucleus, and when the two daughter nuclei were approximately equal in size, staining pattern, and staining intensity^29^. The analysis of abnormal nuclear morphologies (NBUDs or NPBs) was performed on Giemsa-stained BN cells. NBUDs were round or oval protrusions of the nuclear membrane, connected to one of the nuclei whereas NPBs consisted of chromatin segments connecting the two sister nuclei. The data represent the average of at least 3 independent experiments in which a total of 2000–6000 BN cells were analyzed.

### Colony forming assay

To evaluate clonogenic survival, 200–300 untreated cells or cells that had been treated with H_2_O_2_ for 5, 10, or 20 days were plated in T75 culture flasks in triplicate and grown for 15 days. Then, cells were fixed/stained with an aqueous solution containing 0.25% (w/v) crystal violet, 70% (v/v) methanol, and 3% (v/v) formaldehyde, and cell colonies were counted. Only colonies composed of >50 cells, determined by viewing under a bright-field microscope (Axiovert 40 C, Carl Zeiss, Germany), were included in the quantification. The data are reported as the plating efficiency (number of colonies/number of plated cells) and represent the mean of four independent experiments.

### Flow cytometric analysis

Analysis was performed using a Galaxy flow cytometer (DAKO-Agilent Technologies, Glostrup, Denmark) equipped with a 488 nm Argon laser. For cell enumeration, volumetric absolute counting was used. Briefly, for each experimental point, cells were detached, pooled with supernatant culture medium, and centrifuged. The cell pellet was then resuspended in a 5 μg/ml solution of propidium iodide (PI), stained for 5 min at room temperature, and immediately acquired by flow cytometer. PI-positive cells, representing dead cells, were excluded from total live-cell counting. Data were collected from three independent experiments and are reported as average.

For cell cycle analysis, a suspension of ~10^6^ cells from each experimental point was first centrifuged and then fixed by re-suspending the cell pellets in 1 ml of ice-cold 70% ethanol. Subsequently, the fixed samples were incubated for 30 min at 37 °C with 50 μg/ml PI and 150 μg/ml RNaseA. DNA fluorescence was measured with the same Galaxy flow cytometer (DAKO-Agilent Technologies, Glostrup, Denmark) equipped with a 488 nm Argon laser and cell cycle analysis was performed using the Flow Jo^©^ v 7.2.2 software (Tree STAR, OR, USA). Cell cycle data were obtained from three independent experiments and are reported as average.

### Long-term proliferation assessment

Cells were grown for 21 days and counted after 3, 6, 9, 12, 15, 18, and 21 days of culture. At each count, the cells were resuspended and re-seeded at appropriate concentration (100,000 cells/plate). The Cumulative Population Doubling Level (CPDL) was calculated as follows: CPDL = log 2(N_f_/N_0_), where N_f_ is the final cell number and N_0_ is the initial number of seeded cells. The experiment was repeated two times and the data are reported as average.

### Senescence-associated β-galactosidase (SA-β-gal) staining and quantification

For cellular senescence, cells grown on sterile coverslips inside 35 mm Petri dishes were processed using a senescence β-Galactosidase Staining Kit (Cell Signaling Technology, MA, USA) according to the manufacturer’s instructions. The samples were viewed under a microscope and the number of blue-stained (positive) cells vs. β-gal-negative cells was counted in 10–20 different fields of view. Data represent the average of three independent experiments in which a total of 3,029–3,378 cells were analyzed per experimental point.

### RTQ-TRAP assay

The SYBR green RTQ-TRAP assay was conducted as previously described^47^, with minor modifications. Briefly, the reaction was performed with cell extracts (1,000 cells), 0.1 μg of telomerase primer TS, and 0.05 μg of anchored return primer ACX, in 25 μl of SYBR Green PCR Master Mix (Biotools, FL, USA). The primer sequences were those reported by Kim and Wu^48^. Using the Rotor Gene 6000. Thermal cycler (Corbett Life Science, Australia), samples were incubated for 20 min at 25 °C and amplified in 35 PCR cycles with 30 sec at 95 °C and 90 sec at 60 °C (two step PCR). The threshold cycle values (Ct) were determined from semi-log amplification plots (log increase in fluorescence versus cycle number) and compared with standard curves generated from serial dilutions of telomerase-positive (tel+) cell extracts (2,000; 1,000; 500; and 100 cells). Each data point is the average of at least two independent experiments.

### CO-FISH analysis

24 hrs prior to fixation, cells were incubated at 37 °C in the presence of 2.5 × 10^−5^ M 5′-bromo-2′-deoxyuridine (BrdU; Sigma-Aldrich, MO, USA). 5 × 10^−6^ M colchicine was added during the last 5 hrs of incubation. Cells were then collected and metaphase spreads prepared using the protocol described in an earlier section. CO-FISH was performed as previously described^49^ using first a (TTAGGG)_3_ probe labeled with FITC and then a (CCCTAA)_3_ probe labeled with Cy3 (Panagene, Korea). Images were captured with an Axio Imager M1 (Carl Zeiss, Germany) equipped with a Cool Cube 1 CCD camera (MetaSystems, Germany). T-SCEs (Telomere Sister Chromatid Exchanges) were scored only when the double signal was visible with both the Cy3 and FITC probes. The data reported represent the average of 3 independent experiments in which a total of 1,055–4,354 chromosomes were analyzed.

### Combined PML immunofluorescence-telomere FISH

Cells were fixed for 20 min at 4 °C with 4% paraformaldehyde in PBS and permeabilized with 0.1% Triton X-100 in PBS at room temperature. After blocking with 10% BSA at 37 °C for 20 min, cells were incubated with a rabbit polyclonal antibody against PML (H-238: sc5621; Santa Cruz, CA, USA) diluted 1:100 in PBS for 3 hrs at room temperature. After washing with 0.05% Triton X-100 in PBS for 5 min, cells were incubated with Alexa 488-anti-rabbit antibody (Invitrogen, Eugene, OR, USA) diluted 1:300 in blocking solution for 1 hr at room temperature. After immunostainig, the slides were fixed for 2 min in 4% formaldehyde, dehydrated in a series of ethanol washes (70%, 80%, and 100%) and air-dried, prior to hybridization with Cy3-labeled telomeric PNA probe, which was performed as described in the Q-FISH section. After hybridization, slides were washed twice in washing solution (50% formamide, 10 mM Tris pH 7.2, 0.1% BSA) for 15 min, followed by three washes in TBS/Tween 0.08% (0.1 M Tris pH 7.5, 0.15 M NaCl, 0.08% Tween 20). Slides were then dehydrated with an ethanol series and air-dried. Finally, slides were counterstained with 4,6-diamidino-2-phenylindole (DAPI) in Vectashield (Vector Laboratories, CA, USA). Images were acquired using an Axio Imager M1 (Carl Zeiss, Germany) equipped with a 63x objective lens and a Cool Cube 1 CCD camera (MetaSystems, Germany). The data reported represent the average of 3 independent experiments in which a total of 134–382 nuclei per data point were analyzed. The data are reported both as the absolute number of colocalizations per nucleus (Fig. 4F) and as the number of colocalization-positive cells (Fig. 4G). A cell was considered positive when it showed at least three PML/telomere colocalization events.

## Additional Information

**How to cite this article:** Coluzzi, E. *et al*. Transient ALT activation protects human primary cells from chromosome instability induced by low chronic oxidative stress. *Sci. Rep.* **7**, 43309; doi: 10.1038/srep43309 (2017).

**Publisher's note:** Springer Nature remains neutral with regard to jurisdictional claims in published maps and institutional affiliations.

## Supplementary Material

Supplementary Figure S1
