# Supplementary material for: Transient ALT activation protects human primary cells from chromosome instability induced by low chronic oxidative stress
Source: Sci Rep. 2017 Feb 27;7:43309. doi: 10.1038/srep43309 (PMC5327399; doi:10.1038/srep43309)
Supplement: Supplementary Figure S1 [file srep43309-s1.pdf]

Transient ALT activation protects human primary cells from chromosome instability induced by low chronic oxidative stress.

Elisa Coluzzi, Rossella Buonsante, Stefano Leone, Anthony J. Asmar, Kelley L. Miller, Daniela Cimini, and Antonella Sgura

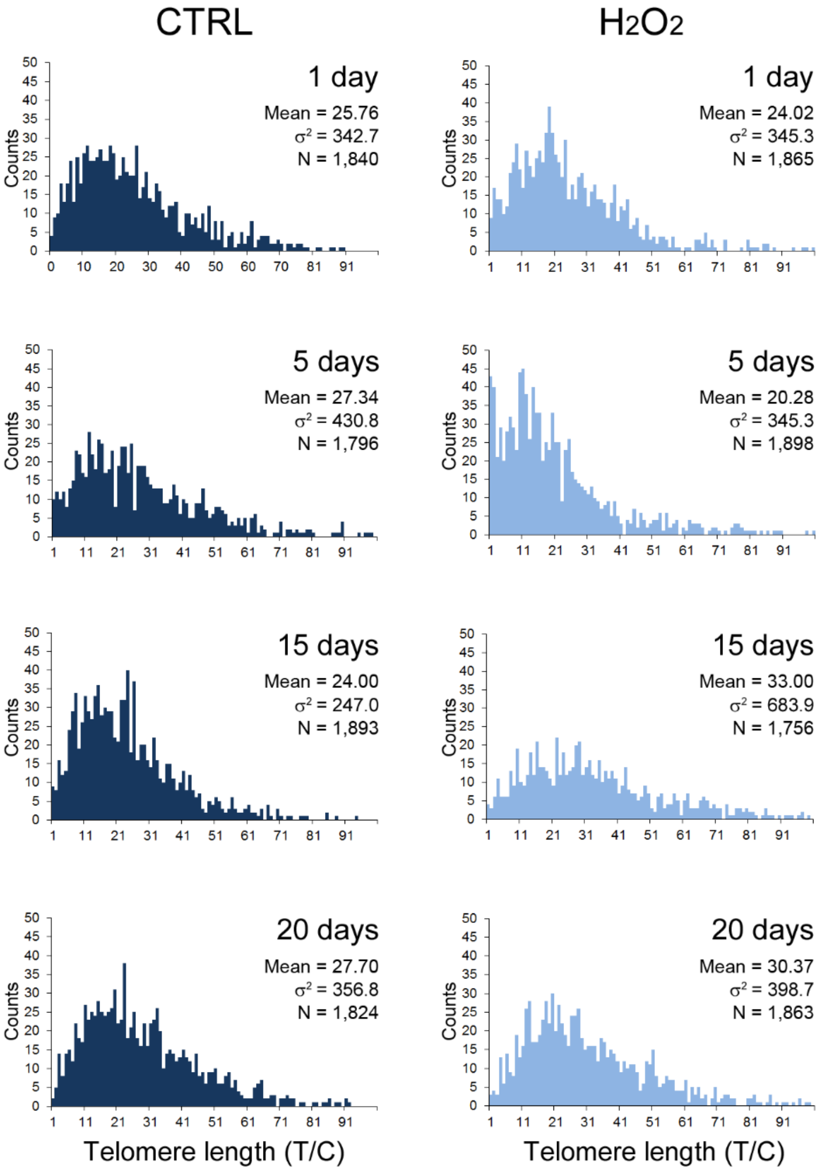

Figure S1. *Telomere size distribution in untreated (CTRL) and H_2O_2-treated cells.* Telomere size data are reported for individual time points (as indicated at the top right corner of each graph) for both untreated (CTRL, left column) and treated samples (H_2O_2, right column). For each data set, the average telomere length (Mean), the telomere length variance (σ^2), and the total number (N) of telomeres analyzed are reported on the right-hand side of the corresponding graph.
